# Fabrication of Low-Power Consumption Hydrogen Sensor Based on TiO_x_/Pt Nanocontacts via Local Atom Migration

**DOI:** 10.3390/nano15151154

**Published:** 2025-07-25

**Authors:** Yasuhisa Naitoh, Hisashi Shima, Hiroyuki Akinaga

**Affiliations:** Core Electronics Technology Research Institute, National Institute of Advanced Industrial Science and Technology (AIST), Higashi 1-1-1, Tsukuba 305-8565, Japan; shima-hisashi@aist.go.jp (H.S.); or akinaga.hiro@ist.hokudai.ac.jp (H.A.)

**Keywords:** hydrogen sensor, local doping, titanium oxide, nanocontact, atom migration

## Abstract

Hydrogen (H_2_) gas sensors are essential for detecting leaks and ensuring safety, thereby supporting the broader adoption of hydrogen energy. The performance of H_2_ sensors has been shown to be improved by the incorporation of TiO_2_ nanostructures. The key findings are summarized as follows: (1) Resistive random-access memory (ReRAM) technology was used to fabricate extremely compact H_2_ sensors via various forming techniques, and substantial sensor performance enhancement was investigated. (2) A nanocontact composed of titanium oxide (TiO_x_)/platinum (Pt) was subjected to various forming operations to establish a Schottky junction with a nanogap structure on a tantalum oxide (Ta_2_O_5_) layer, and its properties were assessed. (3) When the Pt electrode was on the positive side during the forming operation used for ReRAM technology, a Pt nanopillar structure was produced. By contrast, when the forming operation was conducted with a positive bias on the TiO_x_ side, a mixed oxide film of Ta and Ti was produced, which indicates local Ta doping into the TiO_x_. A sensor response of over 1000 times was achieved at a minimal voltage of 1 mV at room temperature. (4) This sensor fabrication technology based on the forming operation is promising for the development of low-power consumption sensors.

## 1. Introduction

As the global demand for sustainable energy solutions intensifies, hydrogen (H_2_) energy emerges as a fundamental alternative to fossil fuels. Its clean combustion and its production of water only makes it an attractive option for reducing carbon emissions. H_2_ gas is used in transportation, power generation, and industrial processes [1,2]. However, the safe implementation of hydrogen technologies necessitates effective monitoring systems. H_2_ gas sensors are essential for detecting leaks and ensuring safety, thereby supporting the broader adoption of hydrogen energy [3,4,5,6]. While various types of hydrogen sensors have been developed [7,8,9,10,11,12,13,14], the metal oxide semiconductor type is one of the sensors that had already been commercialized as a gas alarm because of its simple structure and low energy consumption [15]. Recently, various groups were actively investigating and developing hydrogen sensors utilizing machine learning algorithms to improve gas selectivity [16,17,18]. Moreover, the performance of H_2_ sensors has been improved by incorporating TiO_2_ nanostructures [19,20,21,22,23,24,25]. A recent study demonstrated high-speed H_2_ sensing using a TiO_x_/Pt sensor [26]. Schottky junctions formed at the interface between a thin TiO_x_ layer and a Pt electrode were called TiO_x_ nanocontacts (NCs). The TiO_x_ layer was produced as a native TiO_x_ layer. TiO_x_ NCs achieved H_2_ sensing at room temperature in a dry environment but not in a wet environment. The length and thickness of the TiO_x_ layer of TiO_x_ NCs were small, 20 and 1 nm, respectively; however, its width was approximately 2 μm, indicating that it can be further miniaturized with respect to the width. Functional oxides such as titanium oxide are well known as resistive random-access memory (ReRAM) when sandwiched between metal electrodes. ReRAM is a promising non-volatile memory technology. It operates by inducing local structural changes in the oxide layer with voltage, creating a resistive state for data storage. ReRAM offers high speed, low power consumption, and superior scalability [27,28,29,30,31]. Wei et al. developed an ultra-small, low-power consumption H_2_ sensor by ReRAM using tantalum oxide (TaO_x_) [32]. This ReRAM device was 0.18 µm in size and electrically localized conductive filaments fabricated through a forming operation were even smaller, resulting in low power consumption (0.4 mW). The results indicated H_2_ sensing at room temperature, but the authors did not explore operation in a wet environment. The dimensions of the electrically conductive filaments could be significantly reduced via a forming operation, thus reducing the power consumption of the sensing device. These filament regions were formed in a Ta_2_O_5_ layer, and their resistances were changed by transmitting H_2_ through a metal layer above the Ta_2_O_5_ layer. Furthermore, we recently fabricated planar ReRAMs using exposed conductive filaments [33]. The Ta_2_O_5_ layer has been reported to localize migrated metal atoms in the layer during the forming operation, and in this study, the Ta_2_O_5_ layer was employed to form stable conductive filaments [34]. Pt/TiO_2_/Ti and Pt/TiO_2_/Pt multilayer structures have also been reported to form conductive filaments and function as ReRAMs [28,29]. Combining these techniques could enable low-power consumption H_2_ sensors by exposing localized TiO_x_ NCs.

In this study, several forming operations were performed on TiO_x_ NCs aligned on a Ta_2_O_5_ layer, and the contact characteristics were investigated. During a forming operation with the Pt electrode positively biased, Pt nanopillar structures formed. During a forming operation with TiO_x_ positively biased, a mixed oxide film of Ta and Ti formed. The H_2_ sensing properties of these TiO_x_ NCs achieved a resistance change exceeding 1000 times, even with 1 mV applied at room temperature during the forming operation, resulting in significant power savings.

## 2. Materials and Methods

### 2.1. Fabrication of Sensor Devices

**Figure 1:**
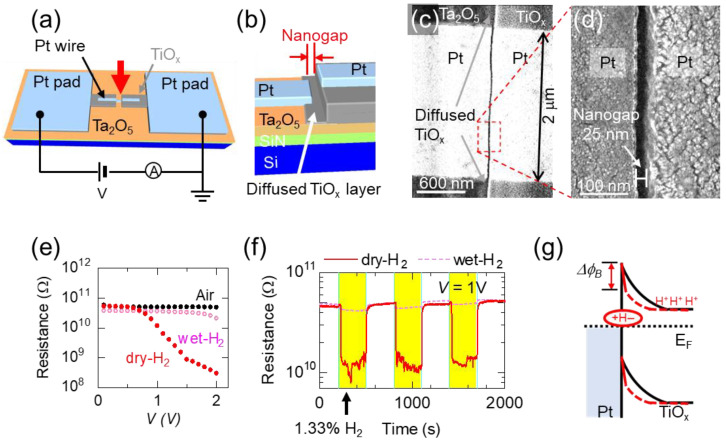
(**a**) Schematic diagram of the fabricated TiO_x_ NC device. (**b**) Magnified image on the red arrows in (**a**). (**c**) Typical FESEM image of fabricated structure. (**d**) Magnified FESEM image of area in red dashed-line box in (**c**). (**e**) Typical resistance–voltage (*R–V*) curves of TiO_x_ NC devices with blowing dry H_2_ (0% RH) and wet H_2_ (88% RH) and without blowing (air). (**f**) Typical time dependence of resistance at 1 V. The solenoid valve was switched on and off in an alternating manner every 300 s to introduce H_2_ gas. The solid and dashed lines indicate dry and wet H_2_, respectively. (**g**) Schematic model and band diagram of devices with (dashed line) and without (solid line) dissociated hydrogen atoms.

Figure 1a,b presents the structure of the TiO_x_ NC devices fabricated in this study. A 10 nm-thick Ta_2_O_5_ layer was deposited via reactive sputtering on a Si substrate coated with a 30 nm-thick SiN layer, which was deposited by low-pressure chemical vapor deposition. Initial and secondary patterns were created using photolithography. Two cycles of shadow evaporation in an electron beam evaporator were used to produce two metal layers. The fabrication procedure is detailed in a previous study [35]. Nanogaps were formed because the metal layer fabricated during the second shadow evaporation was disrupted by the mask at the edges of the first evaporated layer. In the first evaporation, a 26 nm-thick Ti layer was evaporated on to the Ta_2_O_5_ layer. After liftoff, the second patterning step was performed. Subsequently, 1 nm-thick Ti and 13 nm-thick Pt wire ware deposited at certain evaporation angles relative to the substrate during the second evaporation. The size of the gap could be controlled by varying the evaporation conditions. The Pt wires were not connected to Pt pads where probes were placed for the characterization of devices. In this study, without changes in the layer heights, the fabrication conditions led to a gap size of approximately 20 nm. The contact between the left Pt wire and the diffused Ti layer resulted in the formation of TiO_x_ NCs.

### 2.2. Structural and Gas-Sensing Characterization of Sensor Devices

The sensor structures were observed using field-emission scanning electron microscopy (FESEM; Hitachi S5000, Tokyo, Japan). Scanning transmission electron microscopy (STEM) analysis was performed using a JEOL JEM-ARM200F Dual-X (JEOL Ltd., Tokyo, Japan) to evaluate sensor characteristics. The elemental local mappings were investigated using energy-dispersive X-ray spectroscopy (EDX) inside the STEM chamber. A focused ion beam was utilized to fabricate samples for STEM analysis. A schematic of the experimental setup used for gas-sensing characterization is shown in Figure S1. The electrical properties of the fabricated samples were measured using a Keithley 2636 SourceMeter (TEKTRONIX, INC., Beaverton, OR, USA) with a probe station. Measurements were conducted at room temperature (293 K) under ambient conditions (50% relative humidity [RH]). In this study, H_2_ was diluted with balanced dry air (~1.33% H_2_ content; 99.9995% purity for N_2_, O_2_, and H_2_ gases) and balanced dry air were introduced into the solenoid valve at a flow rate of 100 mL/min, which was controlled using the mass flow controllers. Gases were passed through a water-filled gas-washing bottle to increase humidity. Once gases passed through the washing bottle at this flow rate, their RH was 88%, which is typical of wet environments. When a three-port solenoid valve was switched, gas was directed to the fabricated sample through the tip of a micropipette.

## 3. Results and Discussions

### 3.1. Characterization of TiO_x_ NC Device Before Forming Operation

Figure 1c,d shows the FESEM images of the fabricated devices. The diffused Ti layer is connected to the left Pt film. Details of the diffused Ti layer are provided in a previous study [24]. The nanogap size was measured to be 25 nm (Figure 1d), which is slightly larger than the designed size of 20 nm. This discrepancy is discussed in Section 3.5.

### 3.2. Gas-Sensing Properties of TiO_x_ NC Device Before Forming Operation

Figure 1e,f shows the typical resistance–voltage (*R–V*) curves of the fabricated TiO_x_ NC device in dry H_2_ gas, wet H_2_ gas, and ambient air. The curves obtained in dry H_2_ gas exhibited characteristic Schottky conduction behavior [23]. The resistance difference between the dry H_2_ and other gases was measured, indicating that the TiO_x_ NC is applicable to a H_2_ sensor similar to sensors using Pt/TiO_2_ Schottky junctions [23]. Figure 1f shows the typical response of the TiO_x_ NC with and without dry or wet H_2_ gases. Resistance was observed at 1 V. The response of dry H_2_ was distinct. Compared with a previously proposed TiO_x_ NC [26], the present nanocontact exhibited a significantly smaller resistance change, which may be attributed to the difference in the nanogap size. Furthermore, compared with the observed resistance in dry H_2_, wet H_2_ showed minimal sensitivity to H_2_ gas. Although the resistance slightly decreased in Figure 1e, the current change was caused by water adsorption on the nanogap, and the response was to water, not hydrogen [26,36].

Figure 1g shows a schematic band diagram of the TiO_x_ NC sensor. Red and black curves show band structures with and without introducing H_2_ gas, respectively. Upon the introduction of H_2_ gas, the Schottky barrier (Δ*ϕ_B_*) between the Pt and TiO_x_ is reduced due to the dissociative diffusion of hydrogen atoms at the TiO_x_/Pt interface. This reduction in the Schottky barrier is attributed to the formation of dipole layers induced by the diffused hydrogen atoms at the TiO_x_/Pt interface [26]. The current difference in Figure 1e reflects changes in Schottky currents.

### 3.3. Sensor Response Changes of TiO_x_ NC Device After Forming Operation

**Figure 2:**
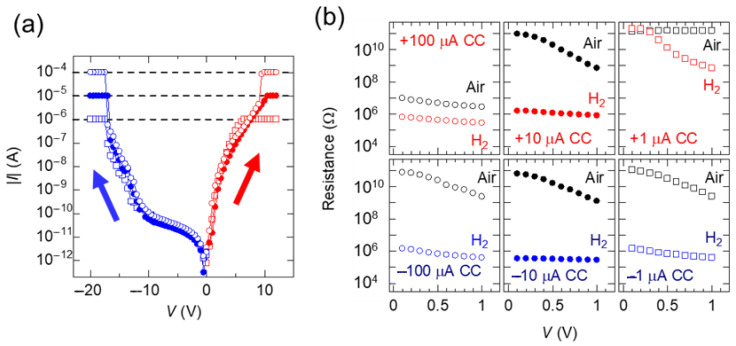
(**a**) Typical current–voltage curves of TiO_x_ NC devices during six forming operations in dry H_2_ environment. (**b**) Typical *R–V* curves of TiO_x_ NC devices after six forming operations with and without dry H_2_.

Figure 2a shows the current–voltage curves of the TiO_x_ NC devices during six forming operations with flowing dry H_2_ gas. The curve with red dots shows the result of applying positive forming voltages from 0 to 12 V to the left side of the electrode (Figure 1a) at a current compliance (*CC*) of +1, +10, and +100 μA. The curve with blue dots shows the result of applying negative forming voltages from 0 to −20 V at a *CC* of −1, −10, and −100 μA. The devices labeled “+10 μA *CC*” were TiO_x_ NC devices fabricated through a forming operation at a *CC* of +10 μA. During the entire forming operation, the observed currents reached the magnitude of the *CC*, indicating that device breakdown could be avoided. The magnitudes of the forming voltages on the negative side were larger than those of the forming voltages on the positive side, which reflects the asymmetric electrical characteristics of the Schottky junction of the TiO_x_ NC.

Figure 2b shows the typical *R–V* curves after the forming operations. Under all forming conditions, the resistances at 1 V in the dry H_2_ environment were consistently lower than that measured in air. The sensor responses in the positive voltage forming operations exhibited large differences at various *CC* values. Therefore, the *CC* values were optimized, thus improving the device performance in this voltage operation. By contrast, in the negative voltage forming operations, the sensor response did not change significantly with the *CC*, suggesting that similar structures formed regardless of the *CC* magnitude. Except in the +1 and +100 μA *CC* cases, the difference in resistance between the measurements in air and dry H_2_ was larger at lower voltages. Thus, the forming operations effectively reduced the required read bias voltage.

### 3.4. Structural Changes in TiO_x_ NC Device After Forming Operation

**Figure 3:**
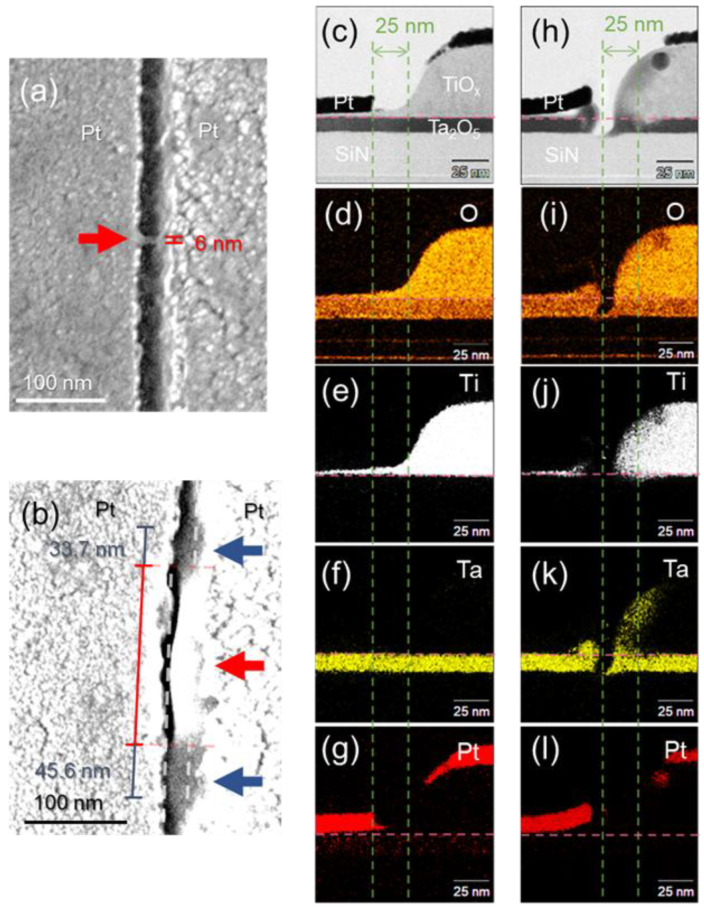
Typical FESEM images of TiO_x_ NC devices at (**a**) +10 μA *CC* and (**b**) −10 μA *CC*. Typical cross-sectional STEM and EDX intensity mapping images of TiO_x_ NC devices at (**c**–**g**) +10 μA *CC* and (**h**–**l**) −10 μA *CC*. EDX intensity mapping images of (**d**,**i**) O, (**e**,**j**) Ti, (**f**,**k**) Ta, and (**g**,**l**) Pt. The purple and green dashed lines indicate the interfaces between TiO_x_ and Ta_2_O_5_ and the positions of the electrode edges before the forming operations.

Figure 3a and Figure 3b show typical FESEM images of the TiO_x_ NC devices at +10 and −10 μA *CC*, respectively. Figure 3a reveals a local 6 nm-wide nanopillar structure (red arrow). On the left electrode, the contrast of the electrode edge varied (arrow), suggesting deformation of the Pt electrode. Moreover, the shapes of their local structural changes differed from those at +100 and +1 μA *CC*, which are shown in Figure S2a,b. The *CC* magnitude and resistance change in Figure 2b corresponded to the amount of structural change. The red arrow in Figure 3b shows these structural changes, such as the curling of the structure and the expansion of the gap width on the right-side electrode. The change at –10 μA *CC* had a larger area than that at +10 μA *CC*. The areas indicated by the blue arrows in Figure 3b indicate areas where scattered structures connected on the upper and lower sides of the curling structure bridging the nanogap. These structural changes (curling and scattering) are also shown in the FESEM images in Figure S2c,d, which show the TiO_x_ NC devices at −100 and −1 μA *CC*, respectively. The curl structures varied depending on the *CC* magnitude, and the scattered structures were similar in size. Considering the relationship between this result and the resistances in Figure 2b, we conclude that the scattered structures are effective for hydrogen response.

### 3.5. Elemental Analysis of TiO_x_ NC Device After Forming Operation

**Figure 4:**
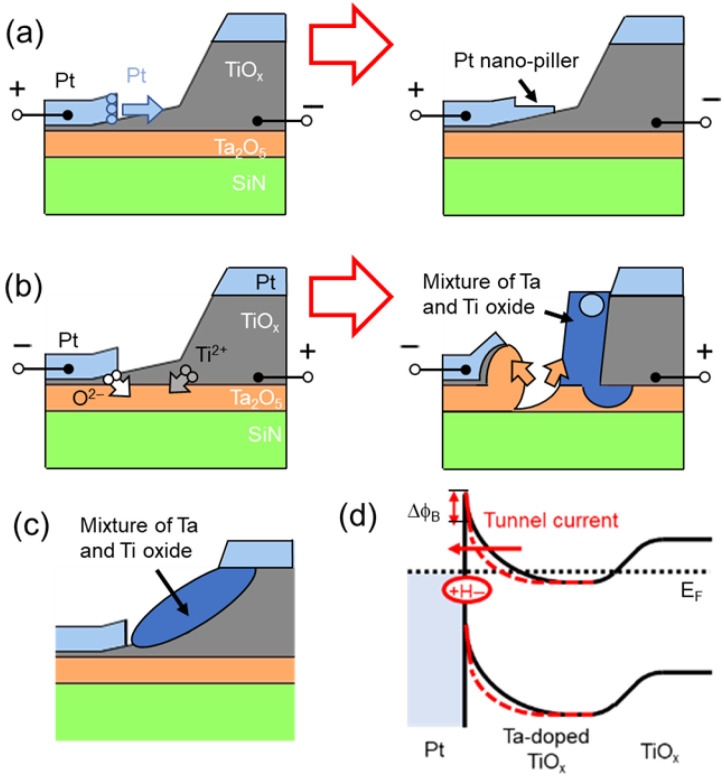
Schematic model of structural changes in TiO_x_ NC devices before and after forming operations at (**a**) +10 and −10 μA *CC* at (**b**) curling and (**c**) scattering positions (after forming). (**d**) Band diagram of TiO_x_ NC devices around scattering position.

Figure 3c shows a typical cross-sectional STEM image of the structural change region after forming operations at +10 μA *CC*. The height of the Ti layers on the right side was ~50 nm, indicating that the evaporated 26 nm Ti layers oxidized and thickened. Figure 3d,e also indicate that the entire Ti layer oxidized and thickened. The Ti layer was oxidized via surface oxidation and self-diffusion from the Ta_2_O_5_ layer [33]. The nanogap width could be extended to 25 nm because of the thickening of the Ti layer, which was the mask for the fabrication of the nanogap [35]. Figure 3f shows that the Ta_2_O_5_ layer was not significantly deformed by the forming operation. Figure 3g shows that the Pt atoms extended from the left-side electrode. When electric fields were applied to the nanogap, metal atoms were pulled up from the surface of the metal electrodes [37]. This observation indicates that a Pt nanopillar structure was created during the forming operation, as shown in the model in Figure 4a, resulting in the fabrication of small local TiO_x_ NC devices.

Figure 3h shows the cross-sectional STEM image of the structural change region around the red arrow in Figure 3b after forming operations at −10 μA *CC*. The Ta_2_O_5_ layer was eroded on the left side and deposited on the right side. The left-side Pt electrode was lifted by the rise in the separated Ta_2_O_5_ layer. The right-side electrode expanded toward the opposite electrode. Figure 3i–k shows that the expanded area consisted of a mixture of Ti and Ta oxide. A comparison of the amounts of Ta and Ti showed that the extended area was likely a Ti-rich oxide layer, resembling a Ta-doped TiO_x_ structure [38,39]. The resistivity of TiO_2_ is significantly reduced by doping it with Ta, and a large resistance change can be induced in the TiO_2_ layer via the forming operation. Figure 4b illustrates a schematic model of structural changes during the forming operations at −10 μA *CC*. During the forming operations, oxygen and Ti ions migrated, and oxygen holes were generated under the edge of the left electrode, suggesting the scattering of Ta oxides [33,40,41,42]. Moreover, Figure 3h reveals a ~10 nm separation between the left and right electrodes, indicating that this area was electrically disconnected and hence did not constitute a conductive path for a H_2_ sensor. The scattered structures (blue arrows in Figure 3b) contained a mixture of Ti and Ta oxide, as shown in Figure 4c, demonstrating the fabrication of a conductive path sized several tens of nanometers for a H_2_ sensor. Local doping is reported to induce changes in Schottky barrier height and band bending [43], it is considered that localized regions were formed where resistance changes occur preferentially in hydrogen reaction. Figure 4d shows a band diagram of the devices around the scattering position. Red and black curves denote the band structures with and without the introduction of H_2_ gas, respectively. Upon introducing H_2_ gas, after the Ta-doped Ti layer formed a highly doped layer in the Schottky junction and ohmic conduction dominated by tunneling conduction, resulting in the linear *R–V* curve at −10 μA *CC* (Figure 2b). The temperature dependence of the *R–V* characteristics before and after forming also supports this result. The results of the temperature-dependent measurements are shown in Figure S3.

### 3.6. Read Voltage Dependence of TiO_x_ NC Device After Forming Operation

**Figure 5:**
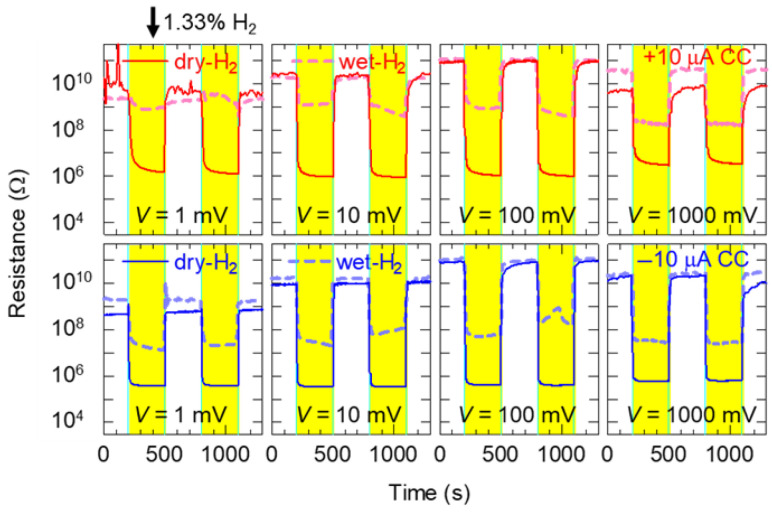
Typical time dependence of resistance at 1, 10, 100, and 1000 mV for TiO_x_ NC devices after forming operations at +10 μA *CC* (upper) and −10 μA *CC* (lower). The solenoid valve was switched on and off in an alternating fashion every 300 s to introduce H_2_ gas. The solid and dashed lines denote dry and wet H_2_ gas, respectively.

Figure 5 and Figure S4 show the response of the TiO_x_ NC devices after the forming operation. Read voltages of 1, 10, 100, and 1000 mV were applied while controlling the humidity during the introduction of H_2_ gas and air, respectively. The yellow regions in Figure 5 and gray regions in Figure S4 indicate the introduction of H_2_ and air, respectively. Here, the high resistance at the read voltages of 1 and 10 mV may be evaluated as a small value because of noise during the current measurement, so the actual resistance change is likely greater than the evaluated values. In Figure S4, although the resistance depended on the humidity response at the read voltage of 1000 mV, this pattern differed at lower read voltages. The humidity dependence of the tunneling current through the nanogap electrodes remained negligible at low bias voltages but showed a relationship between the tunneling current and water vapor pressure at large bias voltages [34]. Therefore, gas sensing at a small read bias voltage can efficiently suppress both tunneling current fluctuations and humidity-induced variations. Table 1 shows the sensor responses (*SR*), sensing times (*T_sen_*), recovering time (*T_rec_*), and power consumption (*P*). The *T_sen_* and *T_rec_* represent the times required for the normalized resistance (ΔR) to reach 0.1 and 0.9 after the introduction and shutting of H_2_ gas, respectively. ΔR and observed resistance (*R_ob_*) are given by

(1) $$∆R=\frac{R_{ob}-R_{H_{2}}}{R_{Air}-R_{H_{2}}},$$

where *R_Air_* and $R_{H_{2}}$ are the resistances just before the flows of H_2_ gas and air, respectively. Power consumption (*P*) is

(2) $$P=V^{2}/R_{H_{2}},$$

where *V* is the read voltage. In the introduction of dry H_2_ gas, clear *SR*s, with resistance changes exceeding 1000, were also observed, even at the read voltage of 1 mV. Moreover, it appears that the smaller the read voltage, the shorter the recovery time. This tendency is consistent with the results of a previous study [24]. These indicate that, in addition to low power consumption, humidity dependence can be effectively suppressed. In contrast, under wet H_2_ gas environments, the resistance changes were smaller than those observed under dry H_2_ gas environments. This suggests the influence of the adsorbed water on the Pt electrode, which worked as a catalyst for hydrogen [20,44,45]. Compared with the TiO_x_ NC devices after forming operations at −10 μA *CC*, those after forming operations at +10 μA *CC* exhibited reduced resistance change magnitudes upon introduction of wet H_2_ gas under applied bias voltage. The former sensors (−10 μA *CC*) showed clearer responses, with SR values exceeding 100 even at a read voltage of 1 mV, even when wet H_2_ gas was flowed toward the sensor.

### 3.7. Sensor Response of TiO_x_ NC Device to Various H_2_ Concentrations After Forming Operation

**Figure 6:**
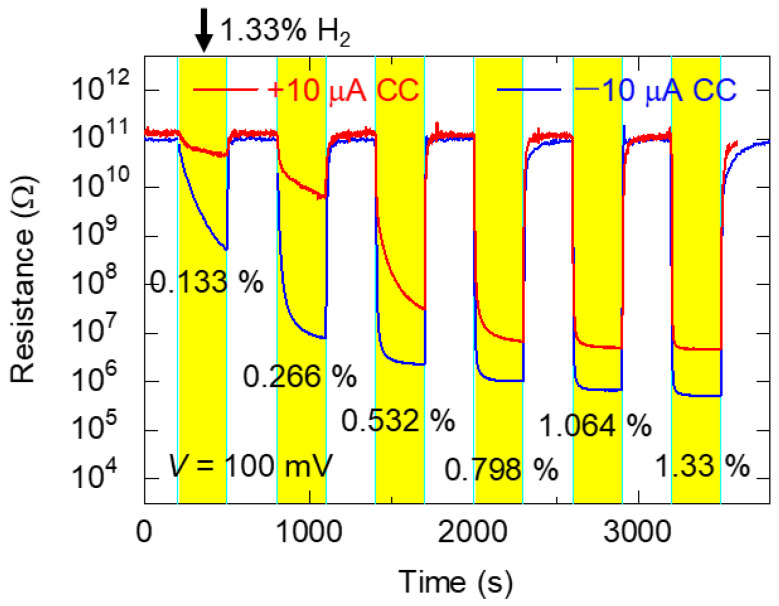
Typical time dependence of resistance at 100 mV for TiO_x_ NC devices after forming operations at +10 μA *CC* (red line) and −10 μA *CC* (blue line) in H_2_ environment at various concentrations. The solenoid valve was switched on and off in an alternating fashion every 300 s to introduce H_2_ gas.

The characteristics of TiO_x_ NC devices after forming operations at +10 and −10 μA *CC* under varying H_2_ concentrations at ambient temperature are depicted in Figure 6. The H_2_ concentration was adjusted by mixing 1.33% H_2_ gas with dry air. The read voltage was 100 mV, which shows the highest *SR* in Table 1. The results demonstrated a correlation between *SR*s and H_2_ gas concentrations, indicating that the sensor may be useful for H_2_ sensing.

## 4. Conclusions

A sample containing a TiO_x_/Pt NC with a nanogap structure on a Ta_2_O_5_ layer was subjected to several forming operations, and its properties were evaluated. During the forming process with the Pt electrode on the positive side, a Pt nanopillar structure was formed, whereas a Ta-doped TiO_x_ layer was produced during the forming process with the TiO_x_ electrode on the positive side. This result indicates that localized doping can be achieved by the application of forming operations. The sensor attained a high resistance ratio, even at a read voltage of 1 mV and low power consumption, because of the forming operations. In particular, sensors with a Ta-doped TiO_x_ layer detected H_2_ even in a moist environment, demonstrating robust environmental performance.

## Figures and Tables

**Table 1:** Humidity and read voltage dependence of sensor response (*SR*), H_2_-sensing time (*T_sen_*), recovering time (*Trec*) and energy consumption (*P*) of TiO_x_ NC devices.

Sample	RH (%)	*V* (mV)	*SR*	*T_sen_* (s)	*T_rec_* (s)	*E* (W)
w/o forming	0	1000	3.60	27.93	18.30	2.10 × 10^−11^
	88	1000	1.10	19.20	14.90	2.00 × 10^−11^
+10 uA CC	0	1	6.03 × 10^3^	1.73	4.98	7.81 × 10^−13^
		10	3.01 × 10^4^	3.07	35.37	1.11 × 10^−10^
		100	9.72 × 10^4^	3.77	150.23	9.47 × 10^−9^
		1000	2.31 × 10^3^	4.40	147.70	3.09 × 10^−7^
	88	1	1.97	1.74	34.32	8.86 × 10^−16^
		10	21.8	1.30	75.07	6.67 × 10^−14^
		100	356	3.97	159.10	2.06 × 10^−11^
		1000	261	2.63	115.00	6.52 × 10^−9^
−10 uA CC	0	1	1.60 × 10^3^	3.02	7.09	2.60 × 10^−12^
		10	2.91 × 10^4^	3.03	40.80	2.86 × 10^−10^
		100	2.07 × 10^5^	2.60	140.43	2.38 × 10^−8^
		1000	3.13 × 10^4^	1.77	120.83	1.57 × 10^−6^
	88	1	104	1.30	24.29	5.10 × 10^−14^
		10	612	1.30	24.37	1.86 × 10^−12^
		100	1.18 × 10^3^	1.33	64.13	9.46 × 10^−11^
		1000	1.17 × 10^3^	2.60	93.50	3.95 × 10^−8^

## Data Availability

Dataset available on request from the authors.

## Supplementary Materials

The following supporting information can be downloaded at: https://www.mdpi.com/article/10.3390/nano15151154/s1, Figure S1: Schematic of experimental setup for gas-sensing characterization, Figure S2: Typical FESEM image of TiO_x_ NC devices at (a) +100 μA *CC*, (b) +1 μA *CC*, (c) −100 μA *CC*, and (d) −1 μA *CC*, Figure S3: Temperature and environment dependence of *R–V* curves and Schottky plots of TiO_x_ NC devices, Figure S4: Typical time dependence of resistance for TiOx NC devices in dry- and wet-air, Figure S5: The long-term stability and repeatability of TiOx NC devices after forming operation, Table S1: Comparison of power consumptions of hydrogen gas sensors.

## Abbreviations

The following abbreviations are used in this manuscript:

AIST	National Institute of Advanced Industrial Science and Technology
ReRAM	Resistive random-access memory
NC	Nanocontact
FESEM	Field-emission scanning electron microscopy
STEM	Scanning transmission electron microscopy
EDX	Energy dispersive X-ray spectroscopy
CC	Current compliance
RH	Relative humidity
SR	Sensor response
