# Supplementary material for: Fabrication of Low-Power Consumption Hydrogen Sensor Based on TiOx/Pt Nanocontacts via Local Atom Migration
Source: Nanomaterials (Basel). 2025 Jul 25;15(15):1154. doi: 10.3390/nano15151154 (PMC12348823; doi:10.3390/nano15151154)
Supplement: Supplementary file 1 [file nanomaterials-15-01154-s001.zip › nanomaterials-3710586-supplementary.pdf]

Supporting information

Fabrication of low-power consumption hydrogen sensor based on TiO_x/Pt nanocontacts via local material migration

Yasuhisa Naitoh[1,*], Hisashi Shima[1], and Hiroyuki Akinaga[1]

[1]Core Electronics Technology Research Institute, National Institute of Advanced Industrial Science and Technology (AIST), Higashi 1-1-1, Tsukuba, Ibaraki 305-8565, Japan; ys-naitou@aist.go.jp (Y.N.); shi-ma-hisashi@aist.go.jp (H.S.); akinaga.hiro@aist.go.jp (H.A.)

[*]Corresponding author:

Yasuhisa Naitoh

Email: ys-naitou@aist.go.jp.

Experimental setup for gas-sensing characterization

Figure S1 shows a schematic of the experimental setup used for gas-sensing characterization. The electrical properties of the fabricated samples were characterized using a Keithley 2636 SourceMeter connected to a vacuum probe station, which maintained a base pressure below 0.01 Pa. The application of bias voltages and switching of the three-port solenoid valve were controlled via LabVIEW 2018 (National Instruments, USA). To prevent pressure from increasing inside the solenoid valve, gas was directed to flow continuously from the gas inlet to the outlet. When the solenoid valve was switched, gas flowed to the sensor device through the tip of a micropipette.

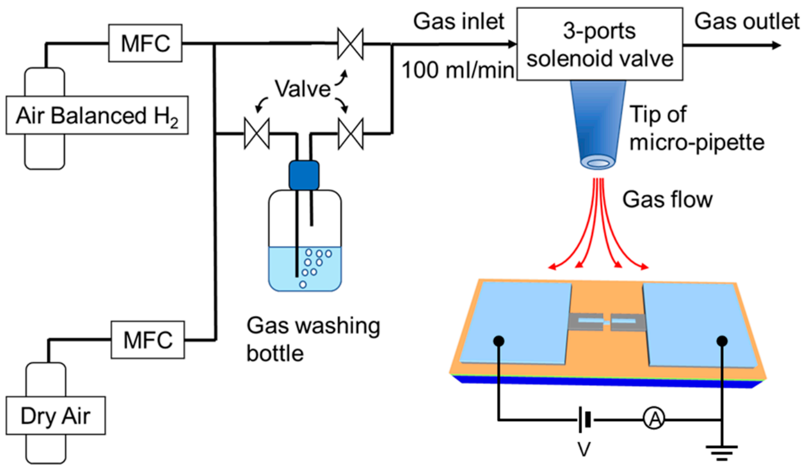

Figure S1. Schematic of experimental setup for gas-sensing characterization. The controls of the solenoid valve and SourceMeter were synchronized using LabView 2018.

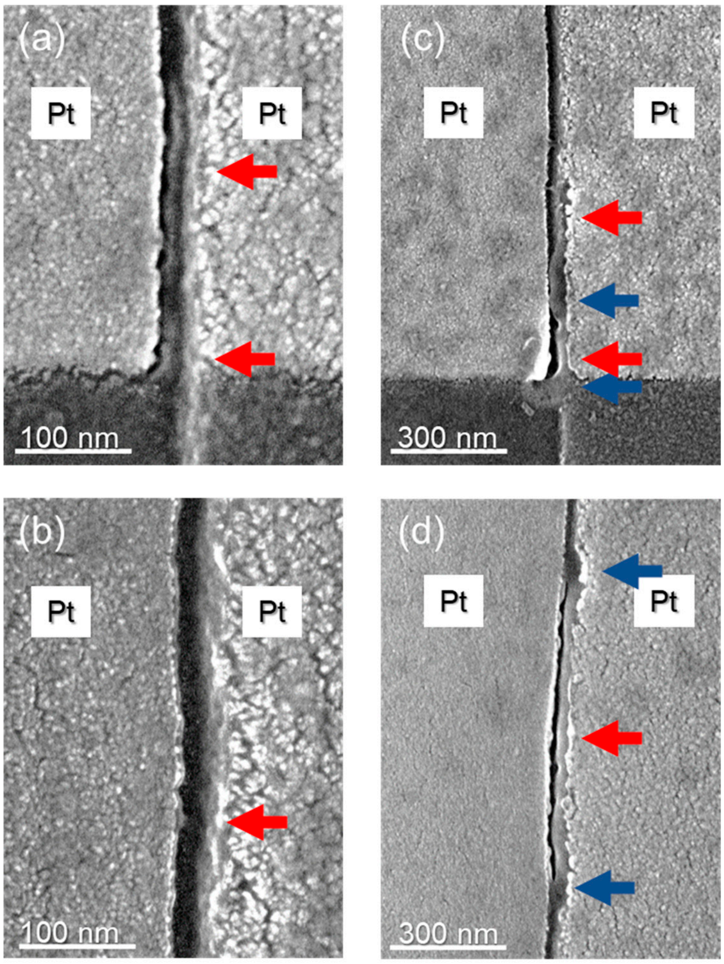

Figure S2. Typical FESEM image of TiO_x NC devices at (a) +100 μA CC, (b) +1 μA CC, (c) −100 μA CC, and (d) −1 μA CC.

Temperature dependence of TiO_x NC device before and after forming operation

To investigate the conductivity mechanism of TiO_x NC devices before and after forming operations, the temperature dependence of conductivity was evaluated. Figure S3a shows the temperature dependences of resistance-voltage (*R-V*) curves of TiO_x NC devices before forming operations, after forming operations at +10 µA CC, and -10 µA CC under vacuum and Ar + H_2 gas environments. H_2 diluted with Ar (H_2 content: ~3.9%; 99.999% purity of Ar and H_2 gases) was introduced into a closed chamber for the Ar + H_2 environment (typical gas pressure: approximately 101 kPa). These *R-V* curves were measured under vacuum and Ar + H_2 because of the difficulty in separating the reaction to the various gases in the air. In previous reports [S1], the TiO_x NC device showed sensitivity to water and oxygen. To eliminate these dependencies, the vacuum probe station was evacuated and filled with Ar + H_2 gas. In the TiO_x NC device without forming operation, a clear decrease in resistance and turn-on voltage with increasing temperature was observed under vacuum conditions (above 2 V), and in Ar + H_2 environment, the decrease in resistance with increasing temperature was also observed. In contrast, for the TiO_x NC devices with forming operations, decreases in resistance were observed with increasing temperature in both vacuum and Ar + H_2, however, the magnitudes of these changes were significantly smaller compared to that of the device without forming operation.

The currents through nanoscale Schottky junction are given by [S2,S3]

$$I = AT^2 exp\left(\frac{a\sqrt{V} - q\Phi}{kT}\right), \text{where } a = \frac{q}{2}\sqrt{\frac{q}{4\pi\varepsilon_i\varepsilon_0 d}}, \qquad \text{(S1)}$$

where A is the effective Richardson constant multiplied by the current injection area, q is the electron charge, Φ is the thermal emission barrier height, V is the applied bias voltage, k is Boltzmann's constant, T is the temperature, ε_i *is* the relative dielectric constant of the TiO_x, ε_0 is the vacuum dielectric constant, and d is the thickness of the molecular dielectric film. Figure S3b displays the plots of $ln(I/T^2)$ versus $1/T$ of TiO_x NC devices measured in vacuum, Ar-H_2, and dry-H_2 environments. The dry-H_2 environment was performed by gas replacements similar to the Ar + H_2. The plots for devices without forming operation clearly show that $ln(I/T^2)$ is linear dependence on $1/T$, and the slope of the line varies with the applied bias voltage. However, the magnitude of the slope changed significantly before and after forming operations, becoming nearly flattened after forming operations, which indicates significant changes in the electrical conduction mechanism. In particular, the flat temperature dependence suggests that leakage currents across the Schottky barrier are dominated by tunnel conduction [S4,S5]. Regarding the differences between the Ar + H_2 and dry-H_2 environment, only small differences were observed for the two devices without

forming and at +10uA CC. In contrast, significant differences were observed for that at -10uA CC, especially above 323 K, where a notable decrease in current was observed. This suggests a distinct reactivity compared to the other two devices, indicating a substantial change in the sensor configuration. This result is consistent with the findings presented in Figure 4c of the main text.

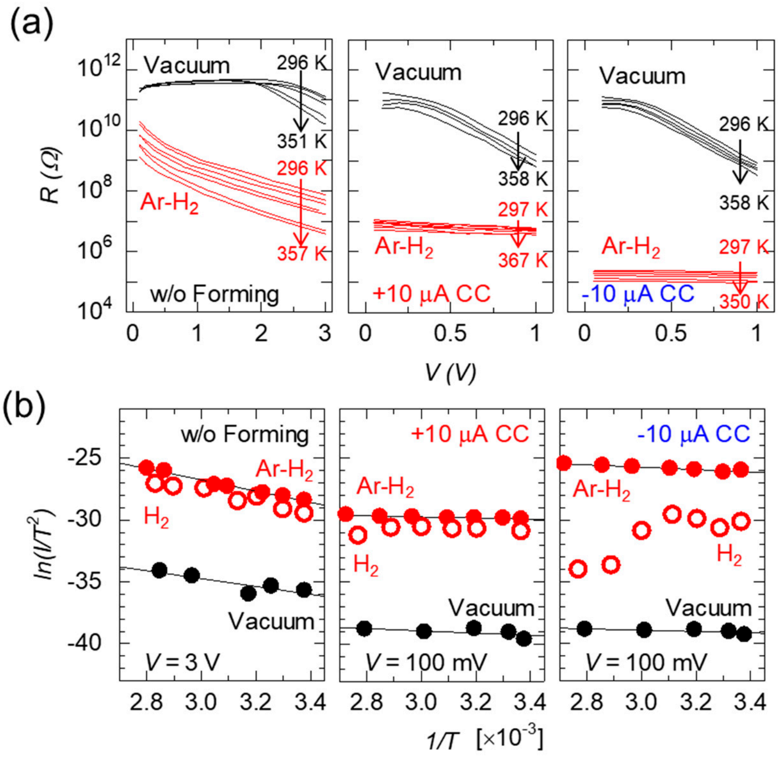

Figure S3. (a) Temperature and environment dependence of *R*–*V* curves and (b) Schottky plots of TiO_x NC devices.

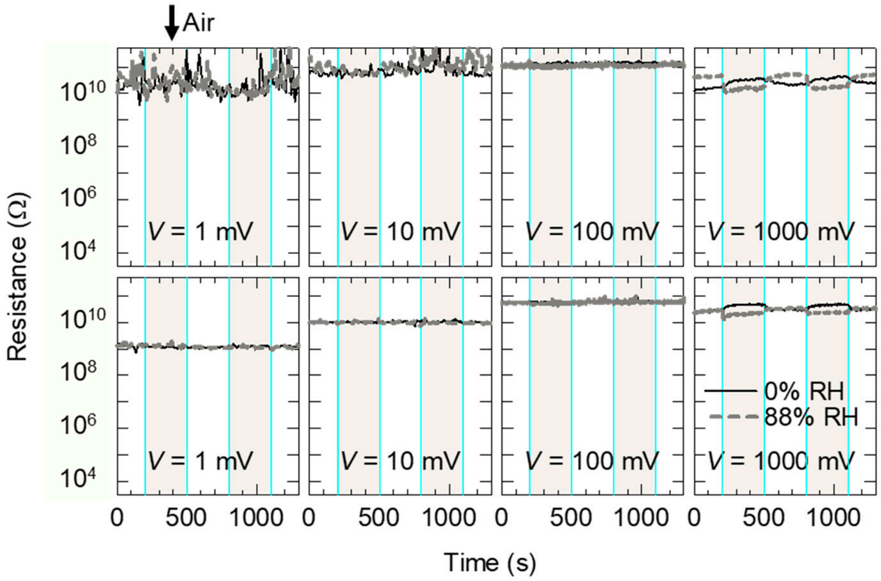

Figure S4. Typical time dependence of resistance at 1, 10, 100, and 1000 mV for TiO_x NC devices after forming operations at +10 μA CC (upper) and −10 μA CC (lower). The solenoid valve was switched on and off in an alternating fashion every 300 s to introduce air. The black solid lines and gray dashed lines indicate dry and wet air, respectively.

The long-term stability and repeatability of TiO_x NC devices after forming operation

Figure S5a shows the long-term stability of TiO_x NC devices in dry H_2. The reading voltage was changed every hour. Sensor resistances were varied from 1000 mV for TiO_x NC devices after forming operations at +10 μA CC and 100 mV for -10 μA CC. The following measurements at 1 mV showed the increase in resistance. These increases are considered to be caused by constant voltage stress, which is a property of conductive filaments in ReRAM, was shown [S6,S7]. This result indicates that a lower reading voltage is preferable for sensor stability. Next, Figures 5b and 5c show 1000 repetitive sensor operations of TiO_x NC devices. Both sensors operated over 1000 times, however the TiO_x NC device after a forming operation at +10 μA CC demonstrated high reproducibility of sensor operations.

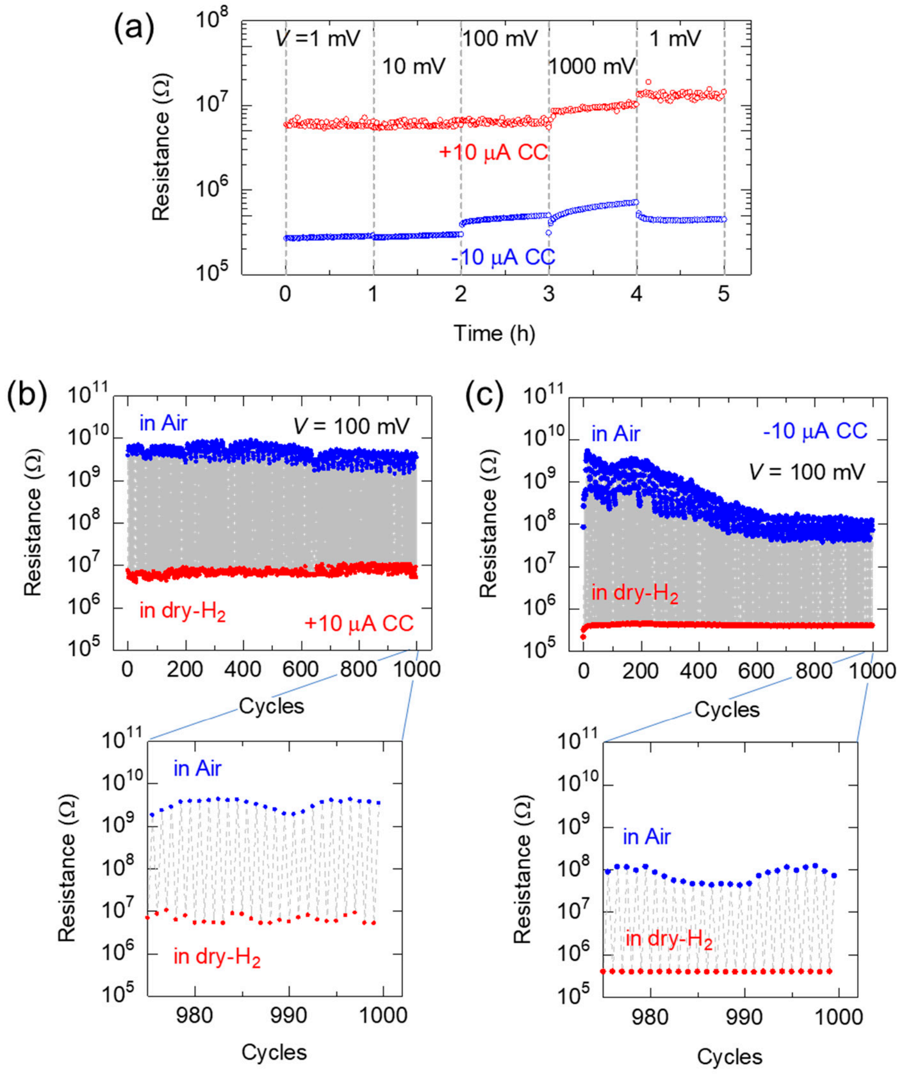

Figure S5. (a) The long-term stability of sensor resistances of TiO_x NC devices after forming operations at +10 μA CC (red open circles) and −10 μA CC (blue open circles). Typical

sensor responces of TiO_x NC devices after forming operations at +10 μA CC (b) and −10 μA CC (c). The solenoid valve was switched on and off in as alternating fashion of 10 and 30 s to control introductions of H_2 gas.

Table S1 Comparison of power consumptions of hydrogen gas sensors.

Sensor type	Heater	Power consumption	Ref.
MEMS-MOX	Micro heater + Pulse heating	<0.1 mW	[S8]
Pt-thin film	Self-heating	0.15 mW	[S9]
Fuel cell-type	Room temperature	0 W	[S10]
MOX	Room temperature	0.4 mW	[S11]
MOX	Room temperature	2.6 pW	This work

References

[S1] Naitoh, Y.; Sumiya, T.; Shima, H.; Akinaga, H. High-speed hydrogen sensor fabricated using a platinum/titanium oxide nanocontact, *Sens. Actuat. B.* **2022**, 371, 132531. https://doi.org/10.1016/j.snb.2022.132531.

[S2] Zhou, C.; Deshpande, M. R.; Reed, M. A.; Jones, II L.; Tour, J. M. Nanoscale metal/self-assembled monolayer/metal heterostructures, *Appl. Phys. Lett.* **1997**, 71, 611. https://doi.org/10.1063/1.120195.

[S3] Araki, K.; Endo, H.; Tanaka H.; Ogawa, T. Multi-Curve Fitting Analysis of Temperature-Dependent I-V Curves of Poly-Hexathienylphenanthroline-Bridged Nanogap Electrodes, *Jpn. J. Appl. Phys.* **2004**, 43, L634. https://dx.doi.org/10.1143/JJAP.43.L634.

[S4] Donoval, D.; Barus, M.; Zdimal, M. Analysis of I–V measurements on PtSi-Si Schottky structures in a wide temperature range, *Sol. State Elec.* **1991**, 34, 1365-1373, https://doi.org/10.1016/0038-1101(91)90031-S.

[S5] Pipinys, P.; Lapeika, V. Temperature dependence of reverse-bias leakage current in GaN Schottky diodes as a consequence of phonon-assisted tunneling, *J. Appl. Phys.* **2006**, 99, 093709. https://doi.org/10.1063/1.2199980.

[S6] Lorenzi, P.; Rao, R.; Prifti, T.; Irrera, F. Impact of the forming conditions and electrode metals on read disturb in HfO_2-based RRAM, *Microelectron. Reliab.* **2013**, 53, 1203-1207. https://doi.org/10.1016/j.microrel.2013.07.043.

[S7] Lorenzi, P.; Rao, R.; Irrera, F. Conductive filament evolution in HfO2 resistive RAM device during constant voltage stress, *Microelectron. Reliab.* **2015**, 55, 1446-1449. https://doi.org/ 10.1016/j.microrel.2015.06.083.

[S8] Dougami, N.; Miyata, T.; Orita, T.; Nakatani, T.; Kakunaka, R.; Taniguchi, T.; Mitsuhashi, H.; Nakao, S. Hot-wire-type micromachined chemiresistive gas sensors for battery-powered city gas alarms, *Jpn. J. Appl. Phys.* **2025**, 64,01SP13. https://doi.org/ 10.35848/1347-4065/ada29c.

[S9] Tanaka, T.; Yanagida, T.; Uchida, K.; Tabuchi, K.; Tatehora, K.; Shiiki, Y.; Nakagawa, S.; Takahashi, T.; Shimizu, R.; Ishikuro, H.; Kuroda, T. Low-Power and ppm-Level Multimolecule Detection by Integration of Self-Heated Metal Nanosheet Sensors, *IEEE Trans. Elec. Dev.* **2019**, 66, 5393-5398. https://doi.org/10.1109/ted.2019.2945932.

[S10] Li, X.; Fei, H.; Zhang, Y.; Zhang, Z.; Zong, H.; Liu, B.; Duan, G.; Luo, Y. Pt/SnO_2-Pd nanoelectrode applied for hydrogen sensor with zero-power consumption and low detection limit, *Sens. Actuat. B.* **2023**, 388, 133848. https://doi.org/10.1016/j.snb.2023.133848.

[S11] Wei, Z.; Homma, K.; Katayama, K.; Kawai, K.; Fujii, S.; Naitoh, Y.; Shima, H.; Akinaga, H.; Ito, S.; Yoneda, S. ; From memory to sensor: ultralow power and high selectivity hydrogen sensor based on ReRAM technology, *IEEE Trans. Electron Devices.* **2018**, 65, 5189–5194. https://doi.org/10.1109/TED.2018.2868081.
